# Genome Editing for Glycogen Storage Diseases

**DOI:** 10.1002/jimd.70249

**Published:** 2026-09-08

**Authors:** Troy von Beck, Raymond Wang, Dwight Koeberl

**Affiliations:** ^1^ Division of Medical Genetics, Department of Pediatrics Duke University School of Medicine Durham North Carolina USA; ^2^ Department of Pediatrics University of California‐Irvine School of Medicine Irvine California USA; ^3^ Division of Metabolic Disorders Rady Children's Health Specialists Orange California USA; ^4^ Department of Molecular Genetics and Microbiology Duke University School of Medicine Durham North Carolina USA

## Abstract

Gene therapy has been developed for several glycogen storage diseases and has advanced into clinical trials. However, the limitations of these gene therapies with regard to stability following treatment early in life have led to the development of genome editing. Early results for genome editing in both glycogen storage disease type Ia and Pompe disease have demonstrated promising efficacy, and proof‐of‐concept studies as well as a clinical trial are underway. These studies will determine whether genome editing fulfills its promise with regard to stably treating glycogen storage diseases early in life.

## 1 Potential for Genome Editing to Address the Limitations of Gene Therapy

Gene therapy for inherited disorders of metabolism has been developed and evaluated in clinical trials; however, the limitations of gene therapy have become apparent and are increasingly being addressed through implementation of genome editing.

The development of gene therapy for glycogen storage diseases has recently been reviewed in detail [1]. While significant progress has been reported, including the launch of clinical trials, limitations of current strategies for gene therapy remain to be addressed. These limitations present obstacles to the successful implementation of gene therapy for inherited disorders of metabolism generally, and largely relate to the adeno‐associated virus (AAV) vectors that have been used frequently for the introduction of a synthetic, therapeutic transgene [2]. Primarily, AAV vector genomes remain as episomes in the nuclei of transduced cells and are lost when those cells subsequently divide. This loss represents a hurdle to the treatment of affected infants early in life due to their potential for multiple cell divisions during normal growth, especially in the liver [3]. Additionally, transgene expression can provoke immune responses, especially when viral enhancers contained in the vectors drive expression in immune cells [4, 5]. Furthermore, AAV capsid proteins provoked T cell responses at higher dosages in clinical trials [6], which eliminated transgene expression in the absence of successful immunosuppression [7]. Finally, complement activation and the associated thrombotic microangiopathy have led to multiple patient deaths at very high vector dosages [8, 9]. These limitations have led to efforts to develop improved genome editing, which will stably transduce patient cells in vivo following administration early in life.

**FIGURE 1:**
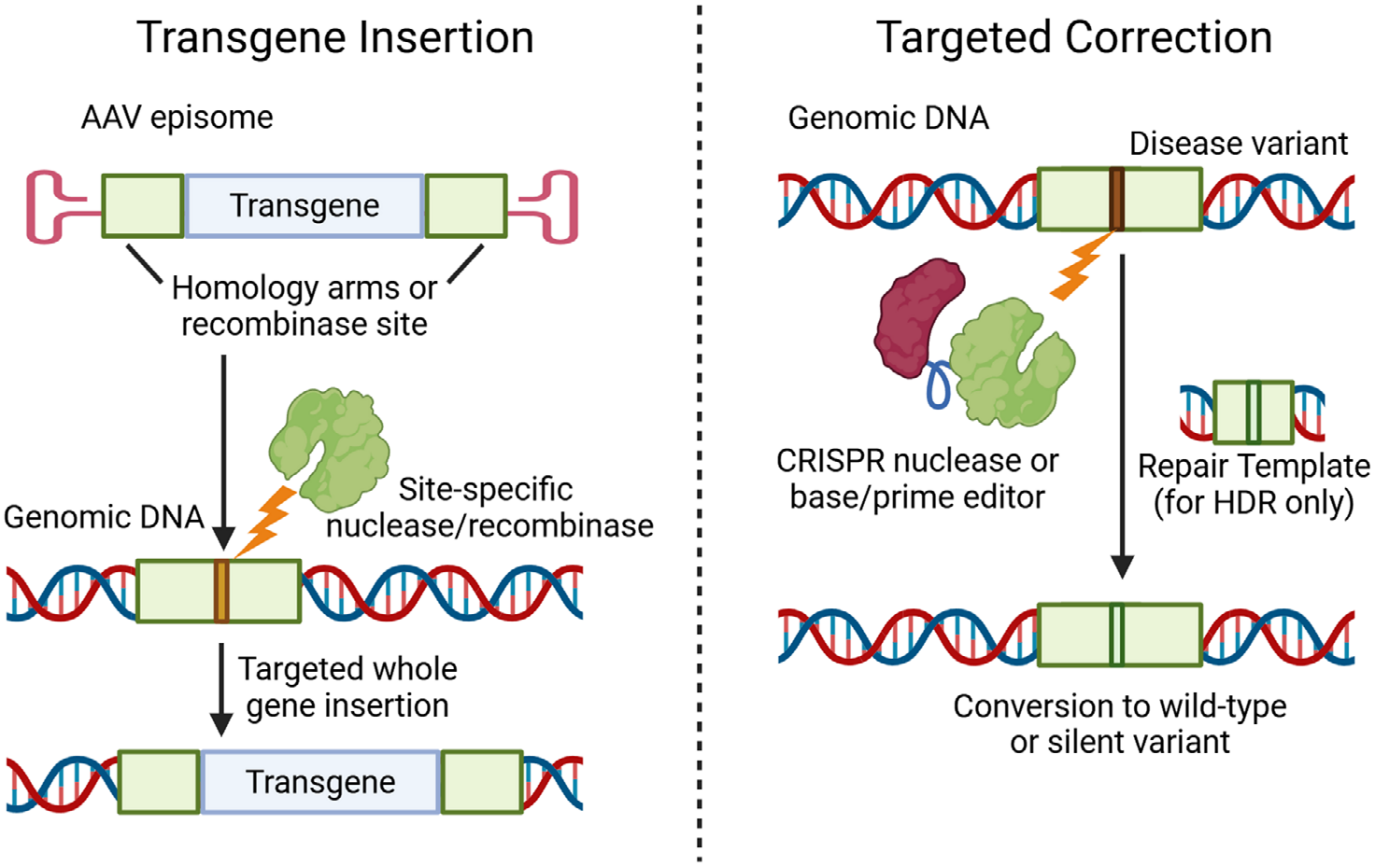
Genome editing strategies. Genome editing for genetic diseases uses either transgene insertion or variant correction. (Left) transgene insertion combines an AAV‐delivered episomal DNA transgene with a targeted nuclease (ZFN, TALEN, CRISPR) or recombinase (LSR). The editing enzyme recognizes specific sequences in the patient genome, initiating a double‐strand break or recombination event that promotes incorporation of the viral episome at a targeted site. (Right) Targeted correction uses a CRISPR‐nuclease or CRISPR‐derived base/prime‐editor to alter a pathogenic gene variant in the host genome. In some designs, a small HDR repair template spanning the gene variant may be supplied via an AAV.

Genome editing can address the limitations of gene therapy, although in vivo demonstration of its potential has been limited to date for glycogen storage diseases (see below). As opposed to gene therapy, genome editing introduces heritable alterations to chromosomal DNA, such that daughter cells are stably corrected [10]. Furthermore, knock‐in and gene correction strategies exploit endogenous promoters to avoid ectopic gene expression, potentially evading immune responses [11]. Indeed, base‐ and prime‐editing represent personalized editing that can correct a pathogenic variant to restore normal gene function (Figure 1; Table 1). However, limitations for genome editing remain, including those related to the vector employed for delivery of genome editing tools, which has often been an AAV vector. Finally, established methods of transgene insertion require cell division to facilitate homology‐directed repair (HDR), which has limited the efficiency of HDR‐dependent genome editing in muscle [12]. Thus, the liver's greater efficiency for HDR and for uptake of vectors makes it much more amenable to genome editing in comparison with other organs [13].

**TABLE 1:** Summary of genome editing approaches.

Editing technology	Efficacy^a^	Safety^a^	Translatability^b^
Gene insertion (nuclease)	Low‐ If vector requires HDR to be functional. Moderate‐ If vector is functional after NHEJ mediated insertion.	Low‐ Requires DSB generation and viral donor template.	High
Gene insertion (recombinase)	Moderate	Moderate‐ Requires viral donor template.	Moderate‐ Limited by feasibility of safe‐harbor site for specific disease indication.
Personalized HDR editing	Low	Low‐ Requires DSB generation and viral donor template.	Low‐ Requires variant specific editor and repair template.
Gene Knockout	High	Moderate‐ If requiring DSB formation High‐Fusing base/prime editor.	Low‐ Only possible where disease is improved by knocking out pathogenic alleles or disrupting secondary disease modifying genes.
Base‐editing	High	Moderate‐ Neighboring bases may be edited.	Low‐ Limited by PAM availability and SNV coverage. Requires unique editor for each variant.
Prime‐editing	Moderate	High	Moderate‐ Requires unique editor for each variant.

^a^ Efficacy and safety vary heavily across genome editor designs and disease indications even within specific editing technology classes. The Low/Moderate/High ratings are intended to provide the reader with an overall impression of the technology across the entire field of genome editing.

^b^ Translatability is a measure of factors affecting clinical implementation not accounted for by efficacy and safety. This category highlights technology limitations that reduce its utility across disease indications and patient variants.

## 2 Status of Clinical Development of Gene Therapy for Glycogen Storage Diseases

Currently, only dietary therapy is available to treat GSD Ia, which consists of supplementation with either uncooked or modified cornstarch. While dietary therapy has succeeded in prolonging the lifespan of people with this condition, it fails to reliably prevent long‐term complications of GSD Ia including hepatocellular carcinoma and end‐stage kidney disease [14]. We and others have successfully piloted AAV‐delivered gene therapy to correct the underlying glucose‐6‐phosphatase (G6Pase) deficiency in GSD Ia. However, the duration of the effect was limited as hepatic AAV vector genome abundance declined rapidly followed by a more gradual loss of biochemical correction [15, 16, 17]. This loss of efficacy from gene therapy presents a hurdle for treatment with gene therapy early in life. Despite the need for early treatment, the clinical trial of gene therapy for GSD Ia (recently approved) enrolled only juvenile and adult patients, and no participant was younger than 8 years old (ClinicalTrials.gov Identifier: NCT03517085). Approaches to this problem have included higher vector dosages [18, 19] and re‐administration of the vector prior to the formation of anti‐AAV antibodies (including in GSD Ia) [20, 21]. Nonetheless, gene therapy loses efficacy and fails to completely reverse the effects of GSD Ia. For example, a recent study in neonatal *G6pc* ^−/−^ mice revealed that the suppression of autophagy associated with GSD Ia was only partially restored in liver [22]. We predict that ignoring these limitations will hamper the progress of liver‐targeted gene therapy for glycogen storage diseases, including GSD Ia and Pompe disease. In Pompe disease the limitations of liver‐targeted gene therapy have been demonstrated [23], although that strategy has been implemented in a clinical trial enrolling adults with late‐onset Pompe disease [24].

## 3 Overview of Genome Editing

To achieve durable disease remission, modern approaches to gene therapy are focusing on techniques to introduce targeted modifications to the chromosomal DNA. Principally, these techniques can be divided into two categories, (1) those relying on targeted nuclease and recombinase enzymes to mediate transgene insertion at a specific locus, and (2) those relying on a targeted DNA modifying enzyme to precisely alter a genomic site (Figure 1; Table 1). Depending on the breadth of activity within a patient population, these techniques can be classified as either “universal” variant agnostic approaches or as “personalized” gene editing restricted to a specific variant. Further, these genetic medicines can be delivered by a variety of means, but most commonly rely on either AAV encoded transgenes and donor templates and/or lipid nanoparticle (LNP) encapsulated mRNA.

When considering whole transgene insertion strategies, the selection of a genomic site for integration is heavily influenced by the sensitivity of the transgene to overexpression and the frequency of dominant negative disease alleles. When neither overexpression nor dominant negative variants are a concern, many approaches opt to use a safe harbor locus for genomic integration. These sites, such as the *AAVS1* and albumin loci offer a safety advantage by virtue of their dispensability for normal function and their repeated characterization across many distinct disease models [25, 26]. However, when an approach requires stricter control over transgene expression or the suppression of dominant negative alleles, it is often advantageous to mediate insertion in the endogenous gene locus. With careful design, this can effectively seize the endogenous *cis*‐regulatory elements for transcription of the transgene cassette and simultaneously halt the transcription of variant alleles.

While AAV delivered transgenes can spontaneously integrate into the genome of transduced cells at a low rate, early studies discovered that targeted nucleases could substantially increase the rate of integration at a specified locus. Historically, we and others have piloted zinc‐finger nucleases (ZFNs) and transcription activator‐like effector nucleases (TALENs) to induce targeted DNA double‐strand breaks (DSBs) which can initiate transgene insertion via either HDR with matching homology arms flanking the transgene or direct ligation to the vector inverted terminal repeats (ITRs) via non‐homologous end joining (NHEJ) [25, 27, 28, 29], both in glycogen storage disease and other inherited metabolic disorders. However, the complex process of engineering a custom ZFN or TALEN for each new DNA target has limited their adoption given the emergence of the highly programmable CRISPR/Cas series of nucleases. By comparison, CRISPR/Cas enzymes do not require target sequence specific protein modifications and are supported by a diverse number of natural and engineered variants that enhance specificity and expand target‐site coverage genome‐wide. The simplicity of changing the target‐site by exchanging only the target‐complementary guide RNA sequence has enabled this system to be adapted to a wide variety of gene therapies for glycogen storage disease and other inherited metabolic disorders that are currently in pre‐clinical stages of development [30, 31, 32].

One major limitation to nuclease‐based insertion strategies is the high frequency of on‐target editing that does not result in transgene integration. This occurs because the generation of the DSBs is functionally separate from the recruitment of the vector genome to the lesion for DNA repair. To overcome this challenge, some studies are now exploring recombinases as a means to permit transgene insertion as the dominant genome editing outcome. While offering higher on‐target activity for genome integration, these enzymes are severely limited by the availability of specific target sites, typically requiring significant optimization of the recombinase and fusion to additional ZFN or CRISPR derivatives to mediate targeted recombination [33, 34, 35]. These considerations position current‐generation recombinases to be uniquely well suited to insertion strategies using safe‐harbor loci, which may be able to justify the high cost of development with their application in transgene integration across multiple genetic diseases.

In addition to the selection of the editing enzyme and usage of donor templates, studies vary by delivery modality for each component. A common design for integrative approaches has been to deliver both the transgene and editing enzyme in separate AAV vectors. This design has effectively treated multiple GSDs in preclinical models but has raised concerns over the long‐term expression of the editing enzyme that could drive additional off‐target effects [29, 30, 31, 32, 36]. This has sparked innovation in hybrid approaches where the transgene and single guide RNA (sgRNA; in the case of Cas9) are delivered in a single AAV vector and the editing enzyme is delivered as in vitro transcribed mRNA encapsulated in a lipid nanoparticle (LNP‐mRNA). Though this approach is mostly limited to liver‐based therapies due to the high liver tropism of current LNP formulations, the added safety from not only limiting the duration of editor expression but preventing genomic insertion of editor encoding DNA sequences makes it an attractive option. In models of both mucopolysaccharidosis type I and hemophilia B, Jin et al. were recently able to achieve transgene HDR rates of 2.1% and 2.6% respectively using the hybrid configuration [37]. Besides limiting the duration of editor expression, LNP‐mRNA is unconstrained by the packaging limits of AAV, allowing for efficient delivery of large fusion proteins without relying on split intein systems or compromising efficiency for the sake of compact editors.

Compared to the gene variant agnostic approach of targeted AAV integration, personalized genome editing promises to offer disease correction with minimal alterations to the patient genome. The first generation of personalized approaches could be considered a derivative of the previously discussed HDR mediated integration, where the donor template only contains the wild‐type gene sequence spanning the patient variant, rather than a whole transgene with flanking homology arms [36]. This design minimizes the donor template size, permitting the in vivo restoration of genes with packaging sizes over the limits of AAV, and even allowing the construction of all‐in‐one AAV vectors containing a repair template, sgRNA, and compact Cas9 for gene editing [38]. However, similar to HDR mediated vector integration, this strategy faces the same challenge of low‐frequency HDR at the target site.

More recent advances focus on the use of engineered Cas9 fusion proteins containing additional protein domains encoding either a DNA‐deaminase/glycosylase (base editors) or a reverse transcriptase (prime editor) to mediate base substitutions and small insertions or deletions at a target‐site. Both prime and base editors have demonstrated advantages for their ability to perform targeted and complex gene modifications with an overall lower risk of genotoxicity (Table 1). Comparing the two technologies, prime‐editing is significantly more flexible, owing to the ability of the reverse transcriptase to not only mediate templated single‐nucleotide changes, but also multiple‐nucleotide changes, deletions, and insertions of up‐to 300 bp [39, 40]. This can make it an optimal candidate for mediating small, targeted changes with substantial improvements in efficiency compared to HDR‐based approaches in non‐dividing tissues. However, this flexibility comes with significantly increased complexity in designing the pegRNA compared to a standard sgRNA and typically an inverse relationship between the complexity of the intended edit and its efficiency [39, 40]. Base‐editing by comparison still has a significant advantage over HDR when introducing a targeted modification but does not cover the full spectrum of SNVs. Base‐editing is also limited by the size of the “editing window” which can result in bystander edits near the target base and inaccessible sites due to the lack of a PAM at an optimal distance from the target. These factors notwithstanding, the feasibility of this approach has been demonstrated by the advancement of a phase I clinical trial for GSD Ia which uses an adenine base editor to correct the R83C variant found in ~33% of Caucasian GSD Ia patientspatient (NCT06735755). In addition to greater efficiency, both approaches typically use nickase variants of Cas enzymes which do not form DSBs and therefore substantially lower the risk of large indel and chromosomal rearrangements. However, it should be noted that while the enzymes do not directly generate DSBs, the following DNA repair processes will stochastically introduce DSBs but at a substantially lower rate [41]. These technologies present a significant opportunity for treating glycogen storage diseases where large segments of the patient population share a single pathogenic variant, such as the c.‐32‐13C>T (so‐called “IVS1”) variant in *GAA* present in 90% of Pompe disease patients, but must be balanced against the cost of developing new editor combinations for each variant. Additionally, while targeted genome editing strategies using nucleases, base editors, and prime editors without delivering a whole replacement transgene are typically restricted to single patient variants, occasionally the unique biology of a given disease permits a “universal” approach by targeting a secondary disease modulating gene. This situation is best demonstrated by the CRISPR gene therapies for sickle cell disease and β‐thalassemia, where the condition can be ameliorated by disrupting the repressor of fetal hemoglobin, whereby the patient specific variants in the beta hemoglobin gene become irrelevant [42, 43, 44].

## 4 Overview of Genome Editing Safety

As with any somatic genome editing modality, comprehensive assessment of Cas‐induced genotoxicity must be performed prior to human therapy. While rigorous in silico design is routinely used to minimize possible mismatched sgRNA/genomic DNA binding and subsequent off‐target editing, off‐target genomic loci nominated by algorithms such as Cas‐OFFinder [45] must be evaluated for unwanted edits. Unbiased analyses such as CHANGE‐seq [46] and GUIDE‐seq [47] should also be performed to identify potential off‐target effects throughout the genome which are not predicted by in silico methods. Long‐read genomic sequencing is suggested if utilizing an editing method that generates DSBs to identify large‐scale deletions or chromosomal rearrangements induced by the DNA repair mechanisms activated by DSBs [48, 49]. To reduce the genomic adverse effects induced by Cas‐induced DSBs, adenine base editors (ABEs) and cytidine base editors (CBEs) were designed which generate a single‐stranded nick and deaminate bases within the editing window [50, 51]. Even then, comprehensive off‐target analyses must be performed to ensure that no off‐target base editing or guide‐RNA independent deamination events occur [52, 53]. Interpretation of these analyses should also consider the duration of nuclease expression, since long‐term expression of Cas9 and guide RNA—as is the case for AAV vectors—is expected to increase the frequency of off‐target edits [54]. As with any nascent technology, the long‐term genotoxic risks of prolonged Cas endonuclease and sgRNA expression are unknown and must be monitored. Finally, long term Cas9 expression may trigger recognition of gene edited cells by cytotoxic T cells, resulting in their removal and a loss of gene therapy efficacy [55].

Since the safety concerns surrounding AAV vectors are well established (comprehensively reviewed elsewhere [56, 57]), this review will focus the discussion of delivery vector safety on the recent clinical data for LNP‐mRNA. Currently, the advancement of LNP technologies into the genome editing space has largely relied on the LP01 ionizable lipid, as well as an undisclosed proprietary lipid developed by Beam Therapeutics. The most common treatment emergent adverse events are transient elevations in liver transaminases and infusion‐related reactions. Intellia Therapeutics has demonstrated safety of gene editing with the LP01 LNP formulation, having treated at least 52 patients with NTLA−2002 for hereditary angioedema and over 650 patients with NTLA‐2001 for transthyretin amyloidosis [58, 59]. Out of these patients, a single 80 year‐old patient with transthyretin amyloidosis and cardiomyopathy experienced a grade 4 elevation in liver transaminases and total bilirubin preceding their death (https://ir.intelliatx.com/news‐releases/news‐release‐details/intellia‐therapeutics‐announces‐third‐quarter‐2025‐financial). Verve Therapeutics has previously terminated their VERVE‐101 clinical trial for heterozygous familial hypercholesterolemia using an ALC‐0307‐based LNP due to serious transient ALT elevation and thrombocytopenia in the 6th patient 4 days after dosing [60]. A reformulated VERVE‐102 product using a GalNAc conjugated LP01 LNP has demonstrated no dose limiting toxicities or severe adverse events across 35 treated patients [61]. Finally, Beam Therapeutics has reported no serious adverse events or dose limiting toxicities across 26 patients dosed with BEAM‐302 for transthyretin amyloidosis using their proprietary LNP formulation (https://investors.beamtx.com/news‐releases/news‐release‐details/beam‐therapeutics‐announces‐compelling‐updated‐clinical‐data). Safety data for patients treated with BEAM‐301 for GSD Ia is not available at the time of writing.

## 5 Genome Editing for GSD Ia by HDR‐Mediated Transgene Insertion

Genome editing has been initiated to correct a mutation or integrate a transgene as a method to stably treat liver metabolic diseases, including GSD Ia [25, 62]. We developed a method using a ZFN‐mediated genome editing method that demonstrated an advantage for genome editing in comparison with gene replacement therapy [25]. Intriguingly, the addition of bezafibrate to induce autophagy during genome editing of *G6pc* ^−/−^ mice more effectively corrected the liver abnormalities of GSD Ia [63]. This was consistent with our hypothesis that the restoration of autophagy will be beneficial in GSD Ia.

**FIGURE 2:**
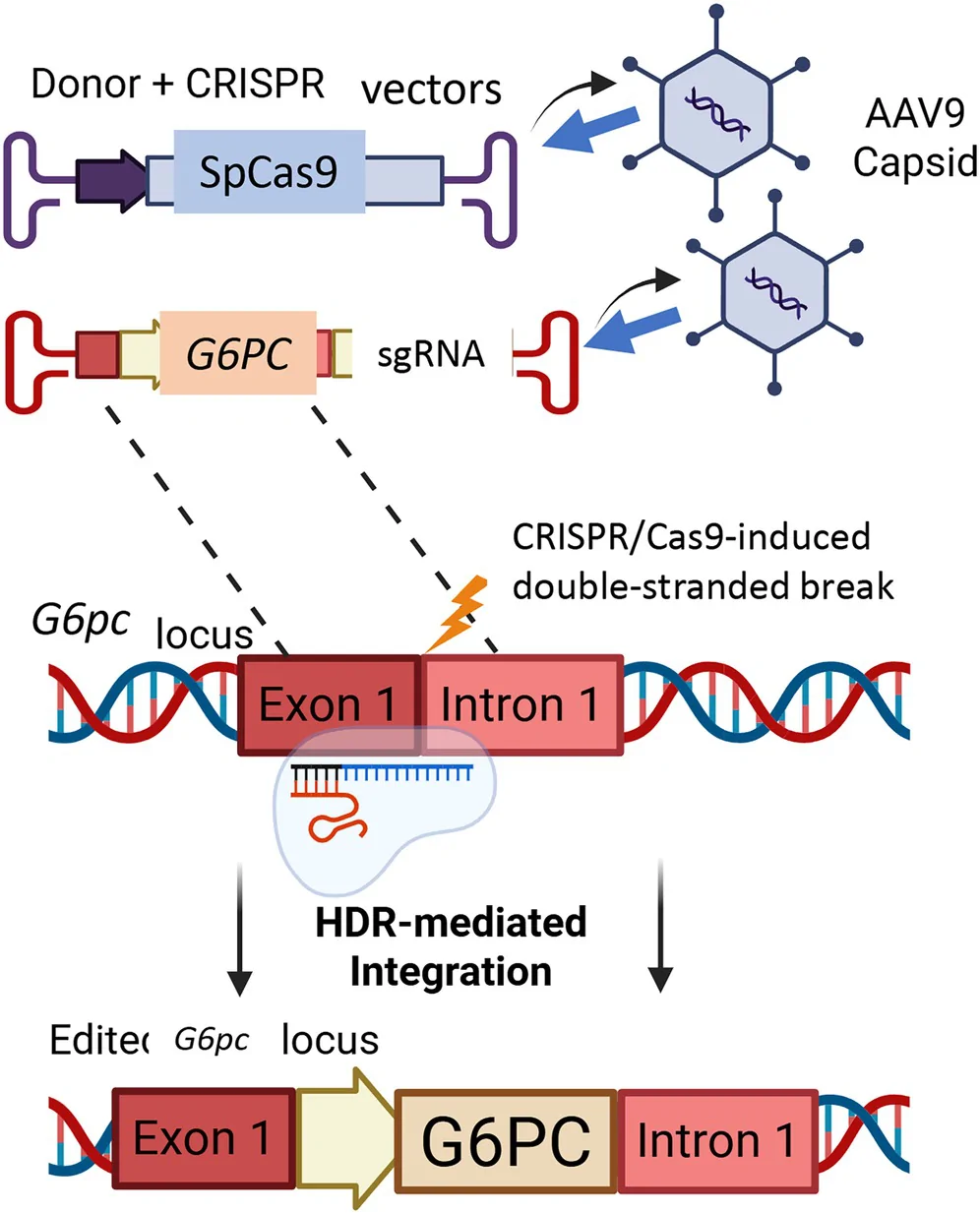
Transgene insertion for GSD Ia. Separate AAV9 vectors are produced encoding (1) the SpCas9 nuclease and (2) the human *G6PC* donor transgene with a minimal *G6PC* promoter flanked by Exon1/Intron1 homology arms and a *G6PC* targeting sgRNA cassette. Upon cellular transduction with both vectors, the SpCas9 nuclease is produced and targeted to the *G6pc* exon 1 intron 1 boundary by the encoded sgRNA. Following CRISPR‐mediated DSB formation, DNA repair may drive HDR between the donor vector homology arms and genomic sequences, leading to permanent incorporation of a functional *G6PC* transgene.

Subsequently we have developed a method of CRISPR/Cas9 mediated genome editing in the canine model for GSD Ia that uses two AAV vectors to deliver the needed components. One vector contains the *Staphylococcus aureus* CRISPR/Cas9 nuclease that cleaves the *G6PC* exon 1/intron 1 boundary, and a second vector delivers a repair template (Donor) to induce HDR and to integrate a functional *G6PC* gene. This strategy inserts a functional *G6PC* cDNA downstream of the *G6PC* gene promoter sequence in the upstream homology arm, which has potential advantages, including: (1) the ability to treat any patient, regardless of the underlying mutation in *G6PC*; (2) early transgene expression from the Donor vector to prevent mortality; (3) safely integrating the transgene in the mutant *G6PC* locus; and (4) driving transgene expression from the endogenous *G6PC* promoter to avoid over‐expression of G6Pase, which could otherwise cause a pre‐diabetic state [64]. This design differs slightly from the design in the mouse study performed later, where a minimal human *G6PC* promoter drove transgene expression, even following transgene integration [63] (shown in Figure 2). We treated three adult dogs and two puppies with GSD Ia to evaluate this method of genome editing [30]. These dogs were also treated with gene therapy to control hypoglycemia from GSD Ia, either prior to genome editing (adults) or following neonatal genome editing (puppies). The necessity of supplemental genome editing reflects the challenges of dietary therapy for large animal models, which correlates with the challenges of compliance with dietary therapy for human patients [14]. Long‐term follow‐up revealed stable genome editing of the canine liver. We confirmed that the HDR‐mediated integration of the Donor transgene is dependent on the CRISPR/Cas9‐mediated nuclease activity, and integration was observed in 0.5 to 1% of *G6PC* genes in the dog liver [30].

The efficiency of genome editing has been evaluated in the neonatal *G6pc* ^−/−^ mouse model, which revealed the benefits of CRISPR/Cas9 to enhance efficacy from genome editing [31]. A short course of bezafibrate was evaluated to enhance genome editing as described [63]. The design was similar to the strategy for the canine study [30], with the exception that the “CRISPR” vector delivers *Streptococcus pyogenes* Cas9 (SpCas9) and the sgRNA is delivered by the “Donor” vector (Figure 2). Both strategies included a *G6PC* promoter in the Donor vector to drive *G6PC* expression from the episomal vector genomes, which treated GSD Ia prior to transgene integration and prevented early mortality. The integrated transgene expressed human *G6PC* from a human minimal *G6PC* promoter in mice with GSD Ia (Figure 2), similar to the ZFN‐mediated method [25]. Mice were treated with either the Donor and CRISPR vectors for genome editing, or the Donor alone for gene replacement (in the absence of the CRISPR vector). Multiple endpoints revealed an advantage for mice treated with the Donor and CRISPR vectors to achieve transgene integration, including: (1) increased survival for mice treated with Donor and CRISPR vectors, in comparison with mice treated with the Donor alone; (2) increased blood glucose during fasting for mice treated with the most effective treatment consisting of high dose Donor and CRISPR vectors with adjunctive bezafibrate therapy [31]. The greatest efficacy was achieved by administering the high dose Donor and CRISPR vectors to drive transgene integration, accompanied by bezafibrate to stimulate autophagy, which had multiple benefits including a high rate of survival and higher blood glucose; furthermore, it achieved 8% of normal G6Pase activity, surpassing the threshold of 3% that prevented HCC formation in GSD Ia [16]. Bezafibrate was also beneficial in the context of ZFN‐mediated genome editing in mice with GSD Ia, by increasing the transduction of hepatocytes and enhancing *G6PC* expression [63]. Furthermore, the stimulation of autophagy increased transduction of hepatocytes with an AAV vector in wild‐type mice, endorsing the potential for general benefits from stimulating autophagy in the context of liver‐targeted gene therapy [65]. Notably, following bezafibrate treatment the integrated transgene could be detected in mice treated with Donor and CRISPR with up to 6% efficiency in the GSD Ia mouse liver [31], much higher than the typical efficiency of approximately 1% in the absence of small molecule enhancement or a selective advantage [30, 36, 66, 67, 68]. This relatively high rate of HDR‐mediated correction, reaching 3% even in the absence of bezafibrate treatment, supports the possibility of positive selection for corrected cells related to the abnormalities of autophagy, apoptosis, and mitochondrial function in GSD Ia hepatocytes [69, 70, 71].

## 6 Personalized Genome Editing for GSD Ia

An alternative approach to genome editing in GSD Ia has been developed to correct a common pathogenic variant in *G6PC*, found in approximately 33% of patients [72]. This variant has been introduced into a mouse model for GSD Ia by inserting a mutant *G6PC* cDNA into exon 1 of *G6pc* such that the cDNA is expressed from the endogenous *G6pc* promoter, generating a humanized model for the common p.R83C variant. Initially, CRISPR‐Cas9 HDR‐mediated genome editing was developed to correct the pathogenic missense variant in *G6PC* and achieved correction in 0.7% of alleles in homozygous affected mice with GSD Ia [36]. Despite the relatively low rate of correction of the variant, mice had no hypoglycemia‐related seizures, > 3% of liver G6Pase activity, and tolerated 24 h of fasting, although blood glucose was lower than normal during fasting. A follow‐up study of a CRISPR/Cas9‐based double‐strand DNA oligonucleotide insertional strategy utilized the non‐homologous end joining repair mechanism to correct the pathogenic variant [73]. When homozygous affected mice were treated at 2 and 4 weeks of age, all survived, in contrast with a single dose at 4 weeks that achieved 15% survival. Mice treated with two doses had ~4% of normal liver G6Pase activity, lacked hypoglycemic seizures, and tolerated 24 h of fasting, although blood glucose was lower than normal during fasting [73]. Correction of the p.R83C variant ranged from 0.1% to 1.4% in the liver [73].

ABE delivered via lipid nanoparticles has been developed to correct the common p.R83C variant (Table 2). The efficiency of base editing is higher than for HDR‐mediated editing, reaching a maximum of 60% in the liver of homozygous affected mice treated at birth and analyzed 3 weeks later, with a mean of 30% correction [78]. However, correction ranged from < 1% to 37% for homozygous affected mice treated at birth or at 3 weeks of age and monitored for 53 weeks. The mean liver G6Pase for mice treated at birth was ~40% of normal, whereas liver G6Pase was ~25% of normal for mice treated at 3 weeks of age. Treated mice had normal blood glucose following 24 h of fasting after 8 weeks, but blood glucose during fasting was low after 53 weeks of monitoring. Intriguingly, a bystander effect was observed with regard to kidney involvement because kidneys for treated mice were less enlarged than for untreated homozygous mice after 53 weeks. Thus, ABE has shown great promise in preclinical models, albeit with wide variability in its efficiency. This strategy for editing the p.R83C variant is currently under evaluation in a Phase 1/2 clinical trial (NCT06735755).

**TABLE 2:** Well‐characterized common pathogenic variants: Responsiveness to genome editing.

GSD type/eponym^a^	Gene/variant	Frequency/population	Genome editing approach		
			Transgene insertion	Prime editing	Base editing
GSD Ia/von Gierke disease [74]	*G6PC1*/p.R83C (c.247C>T)	33%/Caucasian	+	+	+
	*G6PC1*/p.Q347X (c.1039C>T)	18%/Caucasian	+	+	+
	*G6PC1*/c.648G>T	91%/Japanese	+	+	−
	*G6PC1*/c.380_381insTA	50%/Hispanic American	+	+	−
	*G6PC1*/R83H (c.248G>A)	26%/Chinese	+	+	+
	*G6PC1*/c.648G>T	54%/Chinese	+	+	−
GSD Ib [75]	*SLC37A4* /c.1042‐1043delCT	31%/Caucasian	+	+	−
	*SLC37A4*/p.W118R (c.352T>C)	46%/Japanese	+	+	−
	*SLC37A4*/p.G149E (c.446G>A)	29%/Chinese	+	+	+
	*SLC37A4*/p.P191L (c.572C>T)	29%/Chinese	+	+	+
GSD II/Pompe disease [76]	*GAA*/c.‐32‐13C>T	90%/United States	+	+	−^b^
GSD V/McArdle disease [77]	*PYGM*/p.Arg50* (c.148C>T)	70%/Caucasian	+	+	+

^a^ Other GSDs lack well‐documented, high‐frequency variants affecting a large number of patients.

^b^ Unpublished data. The *GAA*/c.‐32‐13C>T cannot be base edited, which we attributed to DNA modifications near the variant.

## 7 Genome Editing for Pompe Disease

Adenine base editing (Figure 1) has been developed for 3 nonsense variants (c.2227C>T/p.Gln743Ter; c.2560C>T/p.Arg854Ter; c.2608C>T/p.Arg870Ter) that cause infantile‐onset Pompe disease (IOPD) [79]. Human dermal fibroblasts obtained from 3 IOPD patients bearing each of these pathogenic variants were nucleofected with ABEmax and variant‐targeting sgRNAs. After 4 weeks, bulk sequencing of ABE‐treated IOPD fibroblasts demonstrated 50%, 41%, and 74% target correction, respectively. Importantly, edited fibroblasts demonstrated GAA enzyme comparable to wild‐type fibroblasts and reduced lysosomal burden measured by decreased LAMP2 protein. Intriguingly, the same group reported initial results for in vivo base editing of the *Gaa* c.2227C>T variant in mice with Pompe disease [80]. The mice were treated with two AAV vectors that used a split intein approach to divide the N‐ and C‐terminal halves of ABE to two viral vectors, which results in trans‐splicing of the expressed proteins in vivo. Treated mice had decreased glycogen content of the heart and skeletal muscle following administration of a total of 1E+14 vg/kg vector particles. These two studies predict that at least a subset of patients with Pompe disease could be treated through adenine base editing; clinical translation of base editing to treat human IOPD is underway.

Genome editing for Pompe disease has great potential for personalized strategies, including base‐ and prime‐editing (Figure 1), although transgene delivery to accomplish genome editing of the heart and skeletal muscle will face limitations related to high vector dosages and associated toxicity needed to achieve genome editing in the muscle, as opposed to the liver. Given the high prevalence of the IVS1 variant in *GAA*, there is a significant opportunity for a personalized gene editing approach. (Table 2). However, neither base nor prime‐editing of the IVS1 variant has been reported, possibly related to DNA modifications that make it inaccessible (unpublished data).

## 8 Potential of Other Glycogen Storage Diseases for Genome Editing

Glycogen storage diseases can be characterized with regard to their organ involvement and pathogenic variants, which dictatesdictate their potential responsiveness to genome editing [1]. The presence of common variants that affect many patients will facilitate personalized genome editing with base‐ or prime‐editing (Table 2). Liver‐targeted genome editing for GSD Ia has advanced more rapidly [30, 36], in comparison with muscle‐targeted genome editing for Pompe disease [79, 80]. This trend will likely continue, making the glycogen storage diseases involving only the liver more amenable to genome editing (e.g., GSD types 0a, Ib, VI, and some cases of type IX) and the glycogen storage diseases involving muscle and heart less amenable (GSD types 0b, III, IV, V, VII, some cases of IX, and others). Glycogen storage diseases with nervous system involvement (Lafora disease, infantile‐onset Pompe disease) could be especially resistant to genome editing due to the challenges of vector‐mediated delivery and genome editing in the brain, although Lafora disease might be responsive to AAV vector‐mediated gene therapy due to the lack of cell division by neurons [81]. Additionally, renal involvement of GSD Ia has been resistant to correction with gene therapy, which will hamper progress toward the correction of renal involvement with genome editing [17]. Overall, genome editing for glycogen storage disease will depend upon the advancement of technology and its application to these individual rare diseases.

## 9 Summary/Conclusions

As the limitations of gene therapy have become generally known, especially for treatment early in life, genome editing has been developed as an alternative approach to achieve long‐lasting efficacy. This trend is apparent in glycogen storage diseases, even as first generation genetic therapies are being evaluated in clinical trials. Preclinical development of both transgene insertion and targeted correction is underway for both GSD Ia and for Pompe disease. The adaptability of CRISPR‐based somatic editing to address pathogenic variants throughout the genome by varying the sgRNA, coupled with the United States FDA promulgation of nucleic acid‐based platform technologies [82], holds great promise for the goal of providing curative therapies for these and many other classical inherited disorders of metabolism.

## Funding

This work was supported by the National Institute of Arthritis and Musculoskeletal and Skin Diseases (R01AR079223).

## Conflicts of Interest

Dr. Koeberl has served as a consultant for Sangamo Therapeutics, Moderna, Takeda, Asklepios Biopharmaceutical (AskBio), and Bayer; has received grant support from Viking Therapeutics, Genzyme Sanofi, Roivant Rare Diseases, and Amicus; and has held equity in AskBio, which is developing gene therapy for Pompe disease. Dr. von Beck has no disclosures. Dr. Wang declares no conflicts of interest.

## Data Availability

Data sharing not applicable to this article as no datasets were generated or analyzed during the current study.
